# Air Pollution and Suicide in Mexico City: A Time Series Analysis, 2000–2016

**DOI:** 10.3390/ijerph16162971

**Published:** 2019-08-18

**Authors:** Claudia Iveth Astudillo-García, Laura Andrea Rodríguez-Villamizar, Marlene Cortez-Lugo, Julio César Cruz-De la Cruz, Julián Alfredo Fernández-Niño

**Affiliations:** 1 Servicios de Atención Psiquiátrica, Secretaría de Salud, Ciudad de México 11410, Mexico; 2 Departamento de Salud Pública, Escuela de Medicina, Universidad Industrial de Santander, Bucaramanga 680002, Colombia; 3 Dirección de Salud Ambiental, Centro de Investigación en Salud Poblacional, Instituto Nacional de Salud Pública, Cuernavaca 62100, Mexico; 4 Departamento de Salud Pública, Universidad del Norte, Barranquilla 081007, Colombia

**Keywords:** suicide, air pollution, confounding factors (epidemiology), Mexico

## Abstract

The association between air pollution and suicide has recently been under examination, and the findings continue to be contradictory. In order to contribute evidence to this still unresolved question, the objective of the present study was to evaluate the association between air quality and daily suicides registered in Mexico City (MC) between 2000 and 2016. Air quality was measured based on exposure to particulate matter under 2.5 and 10 micrometers (µm) (PM_2.5_ and PM_10_, respectively), ozone (O_3_), nitrogen dioxide (NO_2_), and sulfur dioxide (SO_2_), adjusting for weather variables (air temperature and relative humidity), and holidays. To this end, an ecologic time series analysis was performed using a Poisson regression model conditioned by time and stratified by gender and age groups. Models were also generated to explore the lagged and accumulative effects of air pollutants, adjusted by weather variables. The effects of the pollutants were very close to the null value in the majority of the models, and no accumulative effects were identified. We believe these results, in this case, no evidence of a statistical association, contribute to the current debate about whether the association between air pollution and suicide reported in the scientific literature reflects an actual effect or an uncontrolled confounding effect.

## 1. Introduction

Suicide is a global phenomenon that accounted for 1.4% of all deaths worldwide in 2016, 29% of which occurred in low- and middle-income countries, such as Mexico [1]. An increase in the suicide rate in this country has been reported over recent decades [2]. According to national reports, the age-standardized rate was 4.3 suicides per 100,000 inhabitants in 2010, 4.6 in 2012, 5.2 in 2014, and 5.1 in 2016. Eight of every ten suicides are committed by men, with a standardized rate of 8.6 per 100,000 inhabitants in 2016, and 1.9 for women [3]. This poses a significant problem nationally, and young adults between 20 and 29 years of age present the highest rates [3]. Recent reports also document an increase in rates for children and adolescents [4], making suicide a priority problem for public and health policy agendas.

Multiple conditions have been associated with suicide, including mental health disorders, physical disability, significant life events, and socio-cultural stressors such as financial stress, economic crisis, violence, and discrimination, as well as changes in environmental conditions [5]—primarily temperature [6]. More broadly, air pollution is one of the most important risk factors related to adverse effects for human health. An increase in the concentration of air pollutants, especially fine particulate matter, has consistently been shown to be associated with an increase in respiratory and cardiovascular mortality and morbidity in different regions around the world [7].

Recently, air pollution has also been associated with the occurrence of suicides through the hypothesis of neuroinflammation, which states that air pollution could cause an increase in cytokines and reactive oxygen species, and consequently self-aggressive behavior [8,9]. Studies conducted in countries in the northern hemisphere have reported an association between daily concentrations of air pollutants and daily frequency of suicides [10,11,12,13]. Nevertheless, other studies report the absence of this association [14,15]. The differences in the findings present a significant challenge for understanding the effect of air pollution on suicide. In fact, this divergent evidence has been the object of debate about the importance of taking into account possible confounding variables that could be present in the association between pollution and suicide, such as weather and holidays, as well as methodological and analytical considerations [16,17].

Compared with other Mexican cities, Mexico City (MC) has the best air quality monitoring network in the country, in terms of the quality of the information and sufficient data. Furthermore, not only is Mexico city one of the cities with the densest populations nationwide—with 5967 persons per km^2^, much denser than the national average of 61 inhabitants/km^2^ [18,19]—but it is also one of the most polluted cities in Latin America [20]. These characteristics make this an ideal city for observing and studying epidemiological phenomena, such as the topic of this investigation. In addition, air pollution continues to be a problem, and given the multifactorial cause of pollution and the fact that 99.5% of the population is urban, Mexico City residents have been exposed to high levels of criteria pollutants for decades, and more frequently to O_3_, PM_2.5_, and PM_10_, primarily, whose levels have been maintained above the limits allowed in official standards [21]

Given that the evidence continues to be divergent and recognizing the value of replicating the analysis in a highly polluted context where suicides are increasing, we hypothesized if the association between air pollution and suicides was explained by confounding factors. Thus, the objective of the present study was to analyze the association between air pollution and suicides in Mexico City between 2000 and 2016, adjusting for weather variables and holidays as the main sources of confounding. 

## 2. Materials and Methods 

### 2.1. Study Design, Units of Observation and Population

An ecologic, time-series study using daily data related to suicides and air pollutants in MC between 1 January 2000 and 31 December 2016.

### 2.2. Data Collection and Processing

#### 2.2.1. Suicides

Data on the daily suicide count in Mexico City between 2000 and 2016 were obtained from the national vital statistics system of the National Institute of Statistics and Geography (INEGI, Spanish acronym). These vital statistics were constructed based on administrative records from various public offices, by entering data from acts, certificates, and statistical reports [22]. For the denominators, population projections for 2000 to 2016 were used, which were developed by the National Population Council (CONAPO, Spanish acronym), with the population census per municipality, age, and gender in 2005 and 2010 as a basis [23]. Given that this study encompassed only one spatial unit, the denominator was important for taking into account demographic changes in MC over time.

#### 2.2.2. Air Quality and Weather Data 

The hourly air quality data (PM_2.5_, PM_10_, O_3_, NO_2_, and SO_2_) and weather data (air temperature and relative humidity) that were used to generate the exposure variables for the study period (2000–2016) were obtained using the databases from the Meteorology and Solar Radiation Network (REDMET, Spanish acronym) and the Automated Air Monitoring Network (RAMA, Spanish acronym), respectively. Public free access data were available at the website belonging to the Mexico City Ministry of the Environment (SEDEMA, Spanish acronym) (https://www.sedema.cdmx.gob.mx/). Hourly records were used to calculate 24 h averages for each variable analyzed. Data were included only from days that had at least 75% of hourly data measurements for the pollutants. In the case of O_3_, the 8 h moving average was calculated based on that same criterion for the sufficiency of the information. 

Holidays were also considered to be possible confounding variables; this was found in a previous analysis of holidays associated with an increase in the number of suicides registered in Mexico, between 2000–2013 [24]. An indicator variable was generated for New Year (31 December and 1 January), Mother’s Day (10–11 May), Independence Day (15–16 September), and Christmas (24–25 December); previous studies have identified the latter as days that are associated with an increase in suicides in Mexico.

### 2.3. Statistical Analysis

All the air pollutant variables, weather variables, and suicide counts were summarized by central tendency (mean and median) and dispersion (standard deviation and interquartile range). The distribution of daily suicide counts for men and women was evaluated using the dispersion index (VIT) [25] and the asymptotic Böhning test [26]. Based on these, the null hypothesis of equidispersion could not be rejected, and therefore, for the analysis, it was reasonable to assume that the suicides had a Poisson distribution. A Dickey–Fuller test was performed for each time series in order to explore the existence of a unitary root, which is typical of a non-stationary model.

To explore the association between air pollutants and suicides, a Poisson regression model conditioned by time was selected (grouped by day of the week, month, and year). This model enables controlling suicide data by season so that effects can be estimated considering the structure of the correlation that the observations would have when they are generated on the same day of the week, month, and year [27]. By using these conditional time series models, the seasonality of the variables analyzed can be adequately controlled, with a lower computational intensity than other time series models, and with estimation results that are similar to a case-crossover model for analyzing individuals [28].

In order to facilitate interpretation, the daily concentrations of the pollutants were centered by the integer value that approximately corresponded to 20% of the average concentration of the time series. In the case of PM_10_ and PM_2.5_, the values were centered, by convention, on 10 and 5 µg/m^3^, respectively. The Poisson regression models were stratified by gender and age groups (10–29, 30–49, 50–64, and over 65 years). Daily averages for temperature and relative humidity were used to adjust all the models by holidays and weather conditions.

The possibility of lagged effects of the pollutants was then examined, from 1 to up to 7 days before each observation. Fixed Poisson regression models were constructed to explore the accumulative effect of each pollutant, adjusted by weather and holiday variables. In each adjusted model, the susceptible population was adjusted as the exposure variable. The models were evaluated based on the distribution of residuals and goodness of fit tests. All of the analyses were performed using STATA 14 (Stata Corporation, College Station, TX, USA).

## 3. Results

**Figure 1:**
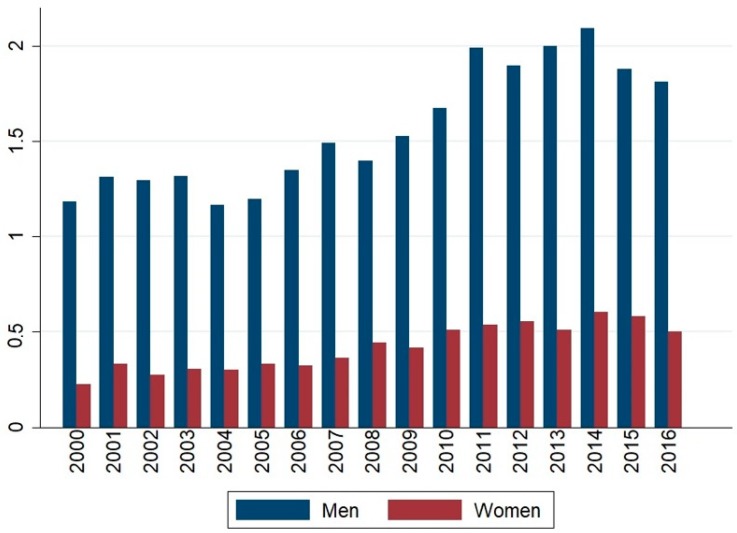
Mean of daily suicides per year in Mexico City 2000–2016.

The average daily suicides registered in Mexico City from 2000 to 2016 was 1.98 (SD 1.49), with a median of 2 and an interquartile range (IR) of 1–3. By gender, the suicide average was 1.56 (SD 1.30) for men, with a median of 1 (IR: 1–2), and 0.42 (SD 0.66) for women, with a median of 0 (IR: 0–1). Figure 1 shows the average number of suicides by sex during the study period, and Table 1 shows daily pollutant concentrations and weather conditions in MC.

Table 2 presents the results of the analysis stratified by gender. All the point estimators obtained for the pollutants are very close to the null value, and the confidence interval includes that in most of the cases. Associations between a decrease in daily suicide count and increased concentrations of NO_2_ are shown, primarily with regard to the effect for men (IRR: 0.96, CI 0.94–0.98). Nevertheless, when stratifying by age group and controlling for the same variables, this effect is seen only for men in the 30–49 age group (Table 3).

Table 4 presents the Poisson regression models that were used to examine the lagged effect for each type of pollutant. Effects near the null value can be seen in all the models, by pollutant and lag time, except for the lagged effect of NO_2_ on day 2, given the increased suicide count among men (IRR 1.03; 1.00–1.06).

## 4. Discussion

This study did not find evidence of an association between concentrations of the pollutants PM_2.5_, PM_10_, O_3_, NO_2_, and SO_2_ and the daily suicide count for men and women in MC from 2000 to 2016 when adjusting for weather variables and holidays. This conclusion is not only based on statistical significance but also on the behavior of the estimators of point associations, and by interval, in all the adjusted models, the majority of which very consistently tended towards the null hypothesis.

There are several possible explanations for the lack of a statistical association between air pollution and the occurrence of suicide in this sample. First, we need to consider factors related to measuring the levels of the air pollutants; in this case, daily mean averages from a network of sites. This can create a random measurement error that could bias the association estimates towards the null value, especially if weak effects exist. It is also important to take into account that MC is an urban area with a very dense population, and high levels of vehicular traffic and industrial emissions generally contain higher concentrations of air pollutants that have little variation over time. This could result in an underestimation of the associations between air pollutants and health results [16], as compared to multi-spatial studies, which by definition would have more variance in pollutant levels, with greater statistical power. Nevertheless, other studies that have been performed at different latitudes and with different levels of contamination also did not find this association [14,15]. And those that that have reported one, such as some studies performed throughout the entire northern hemisphere, studied different pollutants or the associations that were reported were conditioned by time of year [10,12], specific age groups [11,12], or adjusting for other pollutants [13,29].

To summarize, these discrepancies in the scientific literature related to this association can be attributed to randomness, measurement errors resulting from the sources and components of air pollution, confounders, or an actual heterogeneity (modification of the effect) due to differences in the contexts of the different studies, such as weather conditions, cultural backgrounds, socioeconomic factors, and other unmeasured covariables that are associated with suicidal behaviors, as well as the different analytical strategies that could produce errors due to poor specification. In this respect, Kim et al. [13] analyzed this association in multiple locations using a unified modeling strategy in 10 large cities in three countries in northeast Asia: South Korea, Japan, and Taiwan. These cities have similar cultural traditions and have registered relatively high suicide rates (31.0 per 100,000 inhabitants in South Korea, 24.0 in Taiwan, and 17.6 in Japan, compared to a global rate of 11.2 per 100,000, in 2009). While the authors reported that the risk of suicide was greater for higher levels of NO_2_, SO_2_, PM_10_, and PM_10-2.5_, no evidence was found of an association with PM_2.5_, and some of the associations decreased after adjusting for a second pollutant, particularly SO_2_ and NO_2_. 

Taking all of this into account, we believe that the existence of an association between air pollution and the occurrence of suicide, and the actual strength of that association, are far from being clarified. And the debate about whether or not the association could be confounded, which has gone on for some years now, continues to be valid [14,16,17]. In this study, we did not find consistent associations or accumulative effects. And although we cannot discard the possibility of bias or lack of power, we are inclined to think that the association that other studies have found was likely influenced by confounding effects, such as weather variables, holidays, or mental and physical health indicators. It is well known that a weak association could result from an unmeasured confounding variable, or one that was inaccurately or imprecisely measured, or that was poorly specified in the models. In fact, previous evidence from a country that is ecologically and socio-culturally similar suggests that associations initially found in the crude estimations tend to disappear when adjusting for holidays, [12].

Furthermore, it is important to also consider mental health factors, given that roughly 90% of the people who die from suicide have a history of psychiatric disorders, especially mood disorders, those related with substances, anxiety, psychotic disorders, and personality disorders, which commonly affect comorbidity [30]. Therefore, we are inclined to think that mental health factors are a mediating factor in the association between pollution and suicide. For this reason, new studies need to measure the potential effect of air pollution on persons with mental disorders in order to explore whether an interaction exists between the environment and individual susceptibility, which could be hidden when studying the effects on an entire population, such as in the present ecologic study.

In this respect, an investigation by Jin-young Min, Hye-Jin Ki, and Kyoung-bok Min [31] studied a population cohort in South Korea with data from the National Health Insurance Service-National Sample Cohort. They followed 265,749 adults between 2002 and 2013, and found that prolonged exposure to air pollution was associated with a significantly higher risk of death from suicide, adjusted by age, gender, residential area, family income, BMI, exercise, smoking, alcohol consumption, and physical and mental comorbidities, as well as weather variables such as temperature and precipitation, with a hazard ratio (HR) of 3.09 (95% CI: 2.63–3.63) for PM_10_, 1.33 (95% CI: 1.09–1.64) for NO_2_, and 1.15 (95% CI 1.07–1.24) for SO_2_. Nevertheless, when stratifying by mental or physical health conditions, the effect of the pollutants is not present in those who had some type of disorder, and in those who are free of them, it was only maintained for PM10 with an HR of 2.81 (95% CI: 2.26–3.49). This suggests that the association is clearer for those who present with a comorbidity. 

In this regard, another longitudinal study performed in Korea, by Shin, Park and Choi in 2018 [32] with data from 124,205 adults who participated in the Korean Community Health Survey (KCHS), evaluated the association between the risk of presenting different mental health conditions—such as stress, poor quality of life, depressive symptoms, depression, ideation, and attempted suicide—and exposure to high concentrations of PM_10_, NO_2_, and CO between 2012–2013. While their results showed no association between any of the pollutants and attempted suicide after adjusting by confounding factors (age, smoking, alcohol consumption, physical activity, education, marital status, employment, household income, duration of sleep, residence, and medical history), some effects were found for some of the mental health indicators, such as stress, quality of life, and depressive symptoms, especially for those under 65 years of age. This seems to indicate that mental disorders can be present as mediating variables between pollution and suicide.

Buoli et al. [33] described two possible means of association between pollution and mental disorders. The first is an association with the appearance of mental disorders and the exacerbation of symptoms in those presenting mental disorders. The mechanisms for this could be prolonged exposure to a pollutant that can change the epigenetic mechanisms that are responsible for the appearance of psychiatric disorders, or inflammatory and hormonal factors that can cause a mental state to worsen when air pollution rapidly increases. The second means of association could be as a result of changes in the methylation of certain genes, which can trigger the appearance or exacerbation of psychiatric symptoms. In this regard, air pollution could exacerbate psychiatric symptoms in high-risk subjects.

To summarize, in epidemiological terms, rather than a confounding variable, the presence of a mental disorder could be considered a modifying factor that affects susceptibility to the neuropsychiatric effects of pollutants. It is therefore of utmost important to take into account the distribution of mental disorders among people who have committed suicide, without which it would be impossible to extrapolate the effects of pollution on outcomes related with suicide [33]. This is why more recent studies have focused on the association between depression and pollution [33,34,35]. It is also important to consider that roughly 60 to 80% of persons who commit suicide suffer from depression [5].

To summarize the evidence from recent systematic reviews [35], there seems to be a statistically significant but weak association between air pollution and suicides. While it is not possible to discard that air pollution has weak effects on other systems, which do not have the consistency or magnitude of the effects on pulmonary and cardiovascular systems, these findings should be read with caution since these weak effects are particularly susceptible to random errors and bias. In addition, since this topic has only recently begun to emerge, it is possible that the large body of published scientific evidence is reporting a statistically significant association because findings of no association have not been published due to a lack of interest on the part of authors and editors. Thus, this work is aimed at providing evidence of null findings in order to prevent publication bias and contributes to explaining the inconsistencies in the findings.

While it is important to conduct new epidemiological studies, more clinical and experimental studies are also needed in order to better understand the impact of air pollution on mental health, and particularly on biological mechanisms [34], such as longitudinal studies of the effects on different manifestations of suicidal behavior (ideation, planning, and intent). And in general, the need for a causal approach should be considered, in order to determine the actual impact of pollutants on human health outcomes [36].

### 4.1. Limitations

The main limitation of this study was its design. Since it used an ecological approach, this study did not measure individual exposure or covariables that could modify the effects of air pollutants on mental health outcomes. Nevertheless, this is a limitation for the majority of evidence that is available about the effects of air quality on human health.

With regard to the measurements, while there could be a degree of sub-information or incorrect classification of deaths from suicide, in the case of our data, the risk of classification error is lower because each event was verified by a necropsy, and therefore, we believe that incomplete notification did not have a substantial impact. As mentioned previously, while an error in measuring exposure could occur, it would not be differential. Nevertheless, in this case, that could be relevant considering the existence of a weak effect and that this error could bias the associations towards null. 

It is also important to consider the variables that were not evaluated, and that these could be associated with the presence of mental health disorders, especially depression, data about the location of the suicide (whether or not it occurred at home), and the type of suicide. This would help to clarify the effect of external conditions [16] or detect particular scenarios in which an association may exist.

We also need to consider that the data were limited to MC, and therefore, it was not possible to evaluate differences in exposure to air pollutants in urban and rural areas, nor to ensure the exposure time of the subjects, due to the migration in the city. Thus, these findings need to be replicated with other populations and in other geographic regions, nationally as well as in other regions around the world that have different degrees of pollution.

### 4.2. Future Directions

Future investigations should include the study of mental health disorders and compare areas with high mean concentrations of pollutants with those with low pollutant concentrations [34]. They should also analyze relationships with the full range of suicidal behavior since attempted suicide can have different risk profiles than completed suicide [10,16].

In addition, new approaches and technologies for evaluating long-term exposure to air pollution can be used by epidemiological studies. For example, the effects of air pollution can be estimated for a low exposure range by using very large populations (over 1 million adults), for which national cohorts can be an important source of information [31,32]. New technologies, such as GPS, smartphones, and smaller pollution sensors can provide opportunities to evaluate more individualized exposure [37]. While these new technologies and approaches can present new measuring challenges, they can also contribute more evidence about the associations between pollution and mental health.

Lastly, joining the recent debate about the limits of statistical significance for scientific research [38], we adhere to the recommendation about the need to conduct independent analyses in order to see whether the same results are obtained [16]. Ideally, these would use data from studies conducted in different contexts and with different measuring tools in order to obtain a robust replication of the results and truly decrease the degree of uncertainty regarding this association [39].

## 5. Conclusions

Suicide is a multifactorial and preventable phenomenon that is on the increase in Mexico. Therefore, it is crucial to identify the associated variables and propose intervention strategies. This work used a robust statistical approach and controlled the effect of air pollutants by controlling for the main confounding variables, thereby making it possible to contribute high-quality evidence for clarifying the relationship between air pollution and suicide in Latin American countries. Nevertheless, so that the knowledge generated can serve as a basis for a multi-sector approach to preventing suicide, more replications of the results are needed, as well as an epidemiological framework for the analysis. Air pollution is undoubtedly one of the main causes of morbidity and mortality worldwide. And yet, other associations with a lesser magnitude and consistency still need to be clarified, such as those that could affect mental health.

## Figures and Tables

**Table 1:** Daily Concentrations of Pollutants and Weather Conditions in Mexico City, 2000 to 2016.

Variable	Mean	SD	P25	Median	P75	Min	Max
NO_2_ (ppb)	27.75	8.53	21.35	26.70	32.80	9.08	72.19
SO_2_ (ppb)	8.39	7.34	3.25	6.00	11.14	0.63	66.90
O_3_ (ppb)	81.39	28.04	62.10	81.00	99.00	17.00	198.00
PM_10_ (µg/m^3^)	50.47	20.43	34.31	47.66	64.23	9.35	163.96
PM_2.5_ (µg/m^3^)	25.06	10.63	17.44	23.95	31.06	4.38	144.08
Temperature (°C)	16.35	2.40	14.96	16.54	17.80	6.22	23.72
Relative humidity (%)	54.53	14.63	43.77	55.81	65.91	10.79	97.19

SD = Standard Deviation; P = Percentile; Min = Minimum; Max = Maximum.

**Table 2:** Poisson multivariable conditional regression models for daily suicides among men and women in Mexico City, 2000 to 2016.

Pollutants	Men			Women			All		
	IRR	CI 95%	*p*	IRR	CI 95%	*p*	IRR	CI 95%	*p*
NO_2_ (ppb)	0.96	0.94–0.98	0.001	0.99	0.95–1.04	0.936	0.97	0.95–0.99	0.003
SO_2_ (ppb)	0.99	0.99–1.00	0.766	0.99	0.98–1.00	0.532	0.99	0.99–1.00	0.586
O_3_ (ppb)	0.99	0.97–1.01	0.498	0.99	0.97–1.03	0.924	0.99	0.98–1.00	0.513
PM_10_ (µg/m^3^)	1.01	0.98–1.02	0.553	1.01	0.98–1.05	0.505	1.01	0.99–1.02	0.406
PM_2.5_(µg/m^3^)	0.99	0.98–1.01	0.047	1.00	0.97–1.03	0.796	0.99	0.98–1.01	0.952

IRR = Incidence Rate Ratio; CI = Confidence Interval; Note: For each pollutant, the IRR are mean changes in the rates per increase in this 20% of the average (6 ppb for NO_2_, 1 ppb for SO_2_, and 15 ppb O_3_). For PM_10_ and PM_2.5_, the values are centered by convention on 10 and 5 μg/m^3^, respectively. Estimates are adjusted for temperature, humidity, and holidays.

**Table 3:** Poisson multivariable regression conditional models for daily suicides among men and women by age group in Mexico City, 2000 to 2016.

Pollutants	10–29 Years			30–49 Years			50–64 Years			>64 Years		
	IRR	CI 95%	*p*	IRR	CI 95%	*p*	IRR	CI 95%	*p*	IRR	CI 95%	*p*
Men												
NO_2_ (ppb)	0.97	0.94–1.01	0.220	0.93	0.90–0.97	0.001	0.96	0.90–1.035	0.325	0.99	0.91–1.08	0.936
SO_2_ (ppb)	0.99	0.99–1.00	0.884	1.00	0.99–1.00	0.907	1.00	0.98–1.01	0.842	0.99	0.97–1.00	0.272
O_3_ (ppb)	1.01	0.98–1.03	0.388	0.97	0.95–1.01	0.106	0.95	0.95–1.00	0.082	1.03	0.97–1.09	0.344
PM_10_ (µg/m^3^)	1.02	0.99–1.05	0.107	0.98	0.94–1.01	0.251	0.99	0.94–1.06	0.982	1.02	0.95–1.09	0.560
PM_2.5_ (µg/m^3^)	1.01	0.98–1.03	0.613	0.98	0.95–1.01	0.261	0.99	0.94–1.04	0.746	1.02	0.96–1.07	0.485
Women												
NO_2_ (ppb)	1.023	0.96–1.08	0.428	0.99	0.91–1.07	0.827	0.93	0.81–1.07	0.358	0.85	0.68–1.07	0.183
SO_2_ (ppb)	1.00	0.98–1.01	0.868	0.99	0.97–1.01	0.411	0.99	0.97–1.02	0.848	0.97	0.93–1.02	0.344
O_3_ (ppb)	1.00	0.95–1.04	0.965	0.97	0.91–1.03	0.289	1.09	0.99–1.21	0.072	0.95	0.91–1.12	0.587
PM_10_ (µg/m^3^)	1.02	0.97–1.07	0.317	1.01	0.94–1.08	0.794	0.99	0.87–1.12	0.891	0.90	0.75–1.09	0.294
PM_2.5_ (µg/m^3^)	1.02	0.98–1.06	0.227	0.98	0.93–1.04	0.519	0.95	0.86–1.05	0.333	0.97	0.82–1.15	0.768

For each pollutant, the IRR are mean changes in the rates per increase in this 20% of the average (6 ppb for NO_2_, 1 ppb for SO_2_, and 15 ppb O_3_). For PM_10_ and PM_2.5_, the values are centered by convention on 10 and 5 μg/m^3^, respectively. IRR incidence rate ratio, CI confidence interval; estimates are adjusted for temperature, humidity, and holidays.

**Table 4:** Poisson conditional regression models for lagged pollutant effects on daily suicides among men and women in Mexico City, 2000–2016.

Pollutant	Men				Women			All		
	Lag	IRR	95% CI	*p*	IRR	95% CI	*p*	IRR	95% CI	*p*
NO_2_	L1	0.98	0.95–1.00	0.103	1.03	0.98–1.09	0.284	0.99	0.96–1.01	0.341
	L2	1.03	1.00–1.06	0.066	1.00	0.94–1.06	0.972	1.02	1.00–1.05	0.106
	L3	0.99	0.96–1.02	0.444	1.00	0.94–1.06	0.982	0.99	0.96–1.02	0.493
	L4	1.02	0.99–1.05	0.150	0.97	0.92–1.03	0.351	1.01	0.99–1.04	0.397
	L5	1.00	0.98–1.04	0.750	0.98	0.93–1.04	0.512	1.00	0.97–1.03	0.986
	L6	0.99	0.96–1.02	0.623	1.04	0.98–1.10	0.176	1.00	0.98–1.03	0.852
	L7	0.98	0.96–1.01	0.123	0.96	0.92–1.01	0.129	0.98	0.96–1.00	0.039
SO_2_	L1	1.00	0.99–1.00	0.169	1.00	1.00–1.01	0.332	1.00	0.99–1.00	0.431
	L2	1.00	0.99–1.00	0.826	0.99	0.99–1.00	0.221	1.00	0.99–1.00	0.447
	L3	0.99	0.99–1.00	0.030	0.99	0.98–1.00	0.199	0.99	0.99–1.00	0.012
	L4	1.00	0.99–1.00	0.172	0.99	0.98–1.00	0.048	1.00	0.99–1.00	0.035
	L5	1.00	0.99–1.00	0.703	1.00	0.99–1.01	0.959	1.00	1.00–1.00	0.749
	L6	1.00	0.99–1.00	0.168	1.00	0.99–1.01	0.603	1.00	0.99–1.00	0.142
	L7	1.00	0.99–1.00	0.037	0.99	0.98–1.00	0.016	0.99	0.99–1.00	0.003
O_3_	L1	0.99	0.98–1.01	0.514	0.99	0.96–1.03	0.619	0.99	0.98–1.01	0.422
	L2	0.98	0.97–1.00	0.070	1.01	0.98–1.05	0.458	0.99	0.97–1.01	0.204
	L3	0.99	0.97–1.00	0.101	0.98	0.94–1.01	0.156	0.98	0.97–1.00	0.035
	L4	1.00	0.98–1.02	0.787	0.97	0.94–1.00	0.067	1.00	0.98–1.01	0.549
	L5	0.99	0.97–1.01	0.181	0.97	0.94–1.01	0.117	0.98	0.97–1.00	0.056
	L6	0.98	0.96–1.00	0.014	1.00	0.96–1.03	0.794	0.98	0.97–1.00	0.020
	L7	0.99	0.97–1.00	0.179	0.97	0.94–1.00	0.026	0.98	0.97–1.00	0.027
PM_10_	L1	0.99	0.97–1.01	0.164	1.03	0.99–1.07	0.109	1.00	0.98–1.01	0.618
	L2	1.01	0.99–1.04	0.200	1.01	0.97–1.06	0.513	1.01	1.00–1.03	0.151
	L3	1.00	0.98–1.02	0.956	1.00	0.96–1.04	0.978	1.00	0.98–1.02	0.971
	L4	1.01	0.99–1.03	0.440	0.96	0.92–1.00	0.046	1.00	0.98–1.02	0.815
	L5	1.01	0.99–1.03	0.329	0.99	0.95–1.03	0.515	1.01	0.99–1.02	0.572
	L6	1.00	0.98–1.02	0.818	1.03	0.99–1.07	0.124	1.00	0.99–1.02	0.616
	L7	0.98	0.96–1.00	0.022	0.96	0.92–0.99	0.018	0.97	0.96–0.99	0.002
PM_2.5_	L1	0.98	0.96–1.00	0.036	1.02	0.98–1.05	0.375	0.99	0.97–1.00	0.150
	L2	1.01	0.99–1.03	0.424	1.01	0.97–1.04	0.692	1.01	0.99–1.02	0.383
	L3	0.99	0.98–1.01	0.490	0.98	0.95–1.01	0.227	0.99	0.97–1.01	0.241
	L4	1.00	0.98–1.02	0.984	0.98	0.95–1.02	0.369	1.00	0.98–1.01	0.679
	L5	1.00	0.99–1.02	0.768	0.97	0.94–1.01	0.098	1.00	0.98–1.01	0.599
	L6	0.98	0.97–1.00	0.080	1.02	0.99–1.06	0.165	0.99	0.98–1.01	0.361
	L7	1.00	0.99–1.02	0.775	0.98	0.95–1.01	0.180	1.00	0.98–1.01	0.707

Note: For each pollutant, the IRR are mean changes in the rates per increase in this 20% of the average (6 ppb for NO_2_, 1 ppb for SO_2_, and 15 ppb O_3_). For PM_10_ and PM_2.5_, the values are centered by convention on 10 and 5 μg/m^3^, respectively. IRR incidence rate ratio, CI confidence interval; estimates are adjusted for temperature, humidity, and holidays.
